# Balancing Relief and Risk: The Dual Impact of Cannabis on Gastrointestinal Health

**DOI:** 10.34172/mejdd.2025.421

**Published:** 2025-04-30

**Authors:** Amir Mohammad Salehi, Maryam Hasanzarrini, Mohanna Yarahmadi, Fatemeh Shahbazi

**Affiliations:** ^1^Student Research Committee, Hamadan University of Medical Sciences, Hamadan, Iran; ^2^Clinical Research Development Unit of Shahid Beheshti Hospital, Hamadan University of Medical Sciences, Hamadan, Iran; ^3^Modeling of Noncommunicable Diseases Research Center, Health Sciences & Technology Research Institute, Hamadan University of Medical Sciences, Hamadan, Iran

## Dear Editor,

 Cannabis, often referred to as marijuana, is the most widely used illicit drug. It is derived from the cannabis sativa plant and can be ingested, inhaled, or smoked. In the United States, approximately 24 million Americans over the age of 12 are users of this drug.^1^ Research has demonstrated that cannabis use disorder can negatively impact several aspects of human health. One significant consequence is the worsening of mental disorders, including psychosis and schizophrenia.^2^ Additionally, cannabis can lead to various adverse effects on the body, such as structural and functional disorders in the brain, airway inflammation, alterations in reproductive system function, and cardiovascular issues like myocardial infarction, sudden cardiac death, and cardiomyopathy.^3^

 Despite the previously mentioned disadvantages, several studies have highlighted the potential benefits of cannabis treatment. Evidence suggests that Cannabis-based medicinal products (CBMP) can help alleviate symptoms of multiple sclerosis, chronic neuropathic pain, refractory epilepsy in children, anxiety, and insomnia.^4^ In fact, prescribed pharmaceutical-grade CBMP has demonstrated clinical benefits and could serve as a new treatment option for patients.^4^

 Another benefit of medicinal cannabis is its impact on the digestive system. It can be used to treat various digestive disorders, including vomiting, chronic abdominal pain, and inflammatory bowel disease. Several randomized trials have supported the effectiveness of cannabis in alleviating pain associated with irritable bowel syndrome.^5^ Additionally, cannabinoids can help reduce symptoms in patients with gastroparesis and different types of nausea syndromes.^6^ However, Adenusi and colleagues note that the therapeutic use of cannabis can lead to complications such as excessive vomiting syndrome, pancreatitis, and peptic ulcer disease. Additionally, smoking cannabis, especially when inhaled deeply, can cause coughing and irritation of the airways. This chronic coughing and irritation may pressure the lower esophageal sphincter, disrupting normal esophageal function. As a result, this can increase the likelihood of acid reflux and contribute to gastroesophageal reflux disease.^7^

 Because of the conflicting findings regarding the effects of cannabis on the digestive system, it is currently not possible to make a definitive decision about its use in treating digestive disorders. Most available evidence on cannabis stems from cross-sectional studies focused on its recreational use, which are often deemed to be of very low quality. As a result, meaningful conclusions cannot be drawn at this time.^8^ Therefore, more rigorous trials are necessary to obtain reliable results that can help clinicians make informed decisions about the use or non-use of cannabis for treating digestive disorders.
